# Serum tRF-27-FDXXE6XRK45 as a Promising Biomarker for the Clinical Diagnosis in Gastric Cancer

**DOI:** 10.7150/ijms.85180

**Published:** 2023-08-06

**Authors:** Yang Li, Yu Zhang, Xun Li, Xian Li, Xinliang Gu, Shaoqing Ju

**Affiliations:** 1 Department of Laboratory Medicine, Affiliated Hospital of Nantong University, Nantong, Jiangsu 226006, China.; 2 Medical School of Nantong University, Nantong University, Nantong, Jiangsu 226007, China.; 3 Research Center of Clinical Medicine, Affiliated Hospital of Nantong University, Nantong, Jiangsu 226006, China.

**Keywords:** gastric cancer, tRNA-derived small RNAs, tRF-27-FDXXE6XRK45, biomarker, diagnosis

## Abstract

**Objective:** Gastric cancer (GC) has high morbidity and mortality due to inefficient early screening. Therefore, we are searching for more sensitive and specific diagnostic markers for GC. tRNA-derived small RNAs are novel non-coding small RNAs with good abundance and stable presence in body fluids, which may play multiple biological regulatory roles. In this study, we aimed to find a potential biomarker with high accuracy in tRNA-derived small RNAs that can help diagnose GC.

**Methods:** tRF-27-FDXXE6XRK45 was screened as a target molecule by high-throughput sequencing in three pairs of GC tissues. RNA quantitative reverse transcription PCR was conducted to detect the expression levels of tRF-27-FDXXE6XRK45. Agarose gel electrophoresis, Sanger sequencing, cytoplasmic and nuclear RNA isolation assays, gradient dilution experiments, and room temperature and repeated freeze-thaw experiments were used to assess the detection performance of tRF-27-FDXXE6XRK45. Using the chi-square test to analyze the correlation between tRF-27-FDXXE6XRK45 expression levels and clinicopathological parameters. In addition, receiver operating characteristic curves were used to evaluate the diagnostic value of tRF-27-FDXXE6XRK45 in GC.

**Results:** tRF-27-FDXXE6XRK45 expression levels, significantly upregulated in tissues and sera of GC patients and decreased after radical GC surgery, were correlated with the degree of differentiation, depth of tumor infiltration, TNM stage, lymph node metastasis, and nerve/vascular invasion. In comparison with current GC diagnostic markers, tRF-27-FDXXE6XRK45 displayed better efficacy.

**Conclusions:** tRF-27-FDXXE6XRK45, with high diagnostic efficacy, can distinguish GC patients from gastritis patients and healthy donors, suggesting that tRF-27-FDXXE6XRK45 may be a promising candidate as a diagnostic marker for GC.

## Introduction

Gastric cancer (GC), one of the most common malignancies of the gastrointestinal tract 1, has various predisposing factors including Helicobacter pylori infection, age, and a poor diet high in salt and low in vegetables and fruits 2. Globally, GC remains the fifth most common and fourth most deadly cancer despite improvements in diet and medical care 3, 4. Most GC cases are adenocarcinomas originating from the superficial glands or mucosa of the stomach 1. The gold standard for diagnosing GC is upper gastrointestinal endoscopy. However, the early manifestations of GC are rarely noticeable, so costly and invasive upper gastrointestinal endoscopy is not usually performed 5. Furthermore, early screening of GC is inseparable from serum tumor markers, such as carcinoembryonic antigen (CEA), carbohydrate antigen (CA) 199, and CA724, but their positive rates are insufficient 6. Therefore, we are eager to find tumor markers with high sensitivity (SEN) and specificity (SPE) to help screen GC patients.

In recent years, non-coding RNAs (ncRNAs) have been the focus of research and play an essential role in the development of many cancers 7. They are RNAs that do not encode proteins and are classified by length into small ncRNAs and long ncRNAs. Small ncRNAs, including ribosomal RNAs, transfer RNAs (tRNAs), circular RNAs, microRNAs, and Piwi-interacting RNAs, are involved in regulating gene expression 8. Li et al. found that hsa_circ_002059 has the potential as a stable tumor marker for GC 9. Halkova et al. found that miR-27a-3p binding to CA199 significantly improved the diagnostic sensitivity of pancreatic cancer 10. Recently, with the development of high-throughput technologies, more studies have identified an emerging class of non-coding small RNA molecules, tRNA-derived small RNAs (tsRNAs), that play a significant role in cancer.

tsRNAs are small RNA fragments formed by nuclease-specific cleavage of mature tRNA or precursor tRNA, including tRNA-derived fragments (tRFs) and tRNA halves (tiRNAs). Based on the cleavage sites and biogenesis, tRFs can be divided into four major isoforms: tRF-1, tRF-3, tRF-5, and i-tRF. tRF-1, known as 3′ U-tRF, is derived from the 3′ ends of the precursor tRNA cleaved by RNase Z 11. tRF-3 and tRF-5 can be obtained by cleaving the TψC and D loops, while some researchers disagree about whether the production of tRF-3 and tRF-5 is dependent on Dicer 12, 13. i-tRF is usually derived from the internal region of mature tRNAs 14. In addition, tiRNAs are mainly divided into two isoforms: 5′tiRNA and 3′tiRNA. Angiogenin and other RNases can cleave tRNAs to generate tiRNAs under various cellular stress conditions, such as UV irradiation, viral infection, arsenite, oxidative stress, and heat shock 15-18.

Several studies have demonstrated the potential of tsRNAs as novel biomarkers for cancer. For example, Jin et al. found that tRF-Pro-AGG-004 and tRF-Leu-CAG-002 could be potential markers for pancreatic cancer 19. According to Wang et al., tsRNAs such as tRF-Arg-CCT-017 can aid in the diagnosis and prognosis of breast cancer 20. tsRNAs can exert biological effects through multiple pathways. For example, Gebetsberger et al. found that a tRF-5 bound to small ribosomal subunits interfered with the formation of the translation initiation complex of mRNA, thereby inhibiting translation 21. tsRNAs can also bind to AGO proteins to form AGO-tsRNA-mRNA chimeras to silence gene expression 22. In addition, it has been shown that the 5' terminus containing 4 to 5 guanine residues can form RNA G-quadruplexes that replace the translation initiation factor eIF4G/eIF4A in mRNA and thus inhibit translation 23-25. In conclusion, tsRNAs are of high value for research. This study focused on finding a tsRNA that could potentially serve as a GC biomarker.

## Materials and methods

### High-throughput sequencing

We isolated RNA by Trizol and then constructed a small RNA library by the NEBNext® Multiplex Small RNA Library Prep Set for Illumina according to the instructions of the manufacturer. Finally, we operated the HiSeq 2500 SE50 sequencing system to sequence the library.

### Clinical samples

All serum samples in this study, including 130 GC patients, 115 healthy blood donors, 48 gastritis patients, and 42 postoperative GC patients, were obtained from the Department of Laboratory Medicine of the Affiliated Hospital of Nantong University from 2016 to 2021. GC tissues and corresponding paracancerous tissues were collected from 20 of the above GC patients, which were 2-3 centimeters apart. The paracancerous tissues were diagnosed to be free of cancer cell growth and infiltration. All samples collected were promptly stored in a -80°C refrigerator to ensure the quality of the samples for the study. Clinicians diagnosed patients with gastritis in this study, and GC patients were diagnosed by two or more pathologists and did not receive preoperative radiotherapy, chemotherapy, or targeted therapy. By the Declaration of Helsinki, this study respects and safeguards all participants by voluntarily signing the informed consent form. The project was approved by the Ethics Committee of the Affiliated Hospital of Nantong University (ethical review report number: 2018-L055).

### Cell culture

We purchased human GC cell lines (MKN-45, AGS), as well as normal human gastric epithelial cells (GES-1), from the Shanghai Institute of Biological Sciences, Chinese Academy of Sciences (Shanghai, China). All cell lines were cultured in RPMI-1640 medium (Corning, USA) containing 10% fetal bovine serum (Gibco, USA) and 1% penicillin-streptomycin (New Cell, Suzhou, China). The media with cells were deposited in an incubator containing 5% CO_2_ at 37°C under conditions that ensured sterility.

### RNA extraction and RT-qPCR

Serum total RNA was extracted using the Rapid Blood Total RNA Extraction Kit (BioTeke Corporation, Wuxi, China), while total RNA from tissues and cells was extracted using the RNA-easy Isolation reagent (Vazyme, Nanjing, China). We used tRF-27-FDXXE6XRK45 as the target molecule and RNU6B as the internal reference to produce 10 µL cDNA using RevertAid RT Reverse Transcription Kit (Thermo Fisher Scientific, USA) and specific primers (Ribobio Corporation, Guangzhou, China), including tRF-27-FDXXE6XRK45 RT and RNU6B RT. During the process, the prepared 10µL mix was amplified at 42°C for one hour, then inactivated at 70°C for five minutes and stored temporarily at 4°C or long-term at -20°C after completion. Next, real-time quantitative PCR was performed on ABI QuantStudio 5 using a 20µL reaction system consisting of 10µL ChamQ Universal SYBR qPCR Master Mix (Vazyme Biotech Co., Ltd., China), 5µL cDNA, 1µL forward primer, 1µL reverse primer (Ribobio Corporation, Guangzhou, China) and 3µL enzyme-free water. Finally, the expression level of tRF-27-FDXXE6XRK45 was calculated by 2^-ΔΔCT^ method, with the following formula: ΔCT=Ct (target)-Ct (reference) and ΔΔCT=ΔCT (experimental group)-ΔCT (control group). The above steps are performed according to the manufacturer's instructions.

### Nuclear and cytoplasmic RNA isolation assay

MKN-45, AGS, and GES-1 were first collected with trypsin into 1.5 ml EP tubes. According to the instructions of the PARIS™ kit (Thermo Fisher Scientific, USA), 60 µL of nuclear RNA and cytoplasmic RNA were obtained and finally detected by RT-qPCR.

### Gradient dilution experiment

Fifteen serum samples were randomly mixed for RNA extraction and cDNA synthesis, and the cDNA obtained was diluted 10, 10^2^, 10^3^, 10^4^, and 10^5^ times.

### Room temperature and repeated freeze-thawing experiments

Fifteen serum samples were randomly mixed. We placed some of the mixed serum at room temperature (25°C) for 0, 6, 12, 18, and 24 hours, and repeatedly freeze-thawed the other mixed serum at -80°C and room temperature for 0, 1, 3, 5, and 10 times. Then we extracted RNA for detecting the expression of tRF-27-FDXXE6XRK45 by RT-qPCR.

### Statistical analysis

SPSS Statistics version 20.0 (IBM SPSS Statistics, Chicago, USA) and GraphPad Prism v8.0 (GraphPad Software, San Diego, CA) were both utilized for statistical analysis. All data were first tested for normality using GraphPad Prism v8.0 to rule out the possibility of a normal distribution. The Mann-Whitney U test was used to compare two independent groups, and the Kruskal-Wallis H test was used to compare the expression levels of tRF-27-FDXXE6XRK45 in multiple independent groups. The Wilcoxon matched-pairs signed-rank test was used to analyze the expression levels of tRF-27-FDXXE6XRK45 in preoperative and postoperative sera of GC patients, as well as in GC tissues and adjacent tissues. The log-rank test was used to evaluate the significance of survival data between different groups. Youden index was used to define the threshold value of tRF-27-FDXXE6XRK45, and the threshold values for CEA, CA199, and CA724 were set according to the serum tests at the Department of Laboratory Medicine, Affiliated Hospital of Nantong University, at 5 ng/mL, 37 U/mL and 10 U/mL, respectively. The area under the ROC curve (AUC) was used to assess the diagnostic performance of tRF-27-FDXXE6XRK45. The relative expressions of tRF-27-FDXXE6XRK45 from more than 3 independent experiments were shown by the mean ± standard deviation. Differences were considered statistically significant when P < 0.05. *P < 0.05, **P < 0.01, ***P < 0.001, ****P < 0.0001.

## Results

### Screening of tsRNAs and identification of tRF-27-FDXXE6XRK45

**Figure 1:**
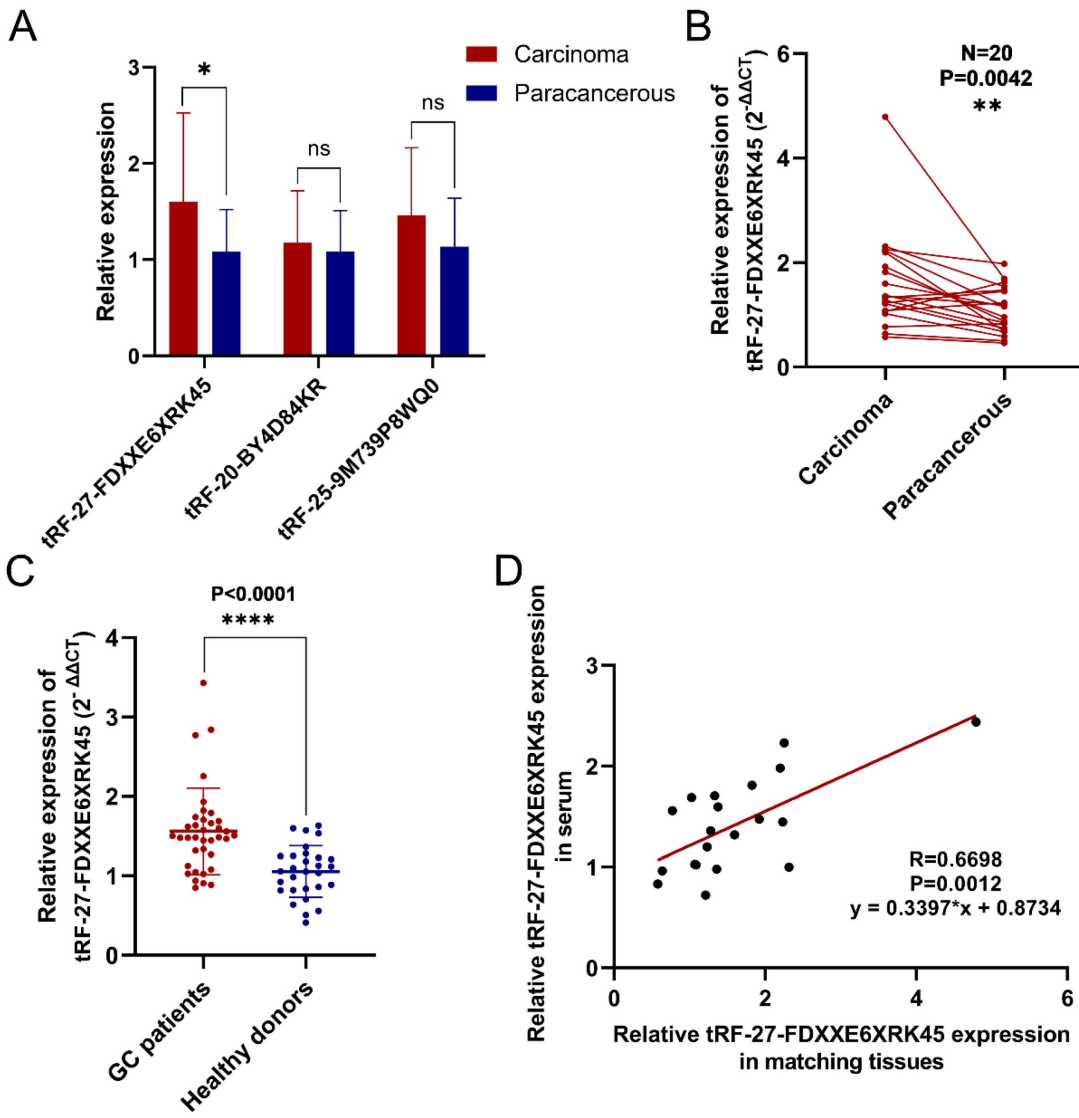
Validation of tRF-27-FDXXE6XRK45 expression in selected GC tissues and sera. **(A)** Relative expression of three highly expressed tsRNAs in 20 pairs of GC tissues. **(B)** The expression levels of tRF-27-FDXXE6XRK45 in 20 pairs of GC tissues and their paracancerous tissues. **(C)** The expression levels of tRF-27-FDXXE6XRK45 in serum samples from GC patients (n=37) and healthy donors (n=29). **(D)** Correlation analysis on the expression levels of tRF-27-FDXXE6XRK45 in 20 GC tissues and corresponding serum samples. *P<0.05; **P<0.01; ***P<0.001; ****P<0.0001

To screen out differentially expressed tsRNAs in GC tissues, we performed high-throughput sequencing on three pairs of GC tissues and their corresponding paraneoplastic tissues. According to the sequencing results, we screened three up-regulated (log2(fold change)>1, P<0.05) tsRNAs (tRF-27-FDXXE6XRK45, tRF-20-BY4D84KR, tRF-25-9M739P8WQ0) to validate them. The results showed that tRF-27-FDXXE6XRK45 was significantly highly expressed in GC tissues **(Fig. 1A)**. Subsequently, we collected GC tissue samples from 20 patients for RT-qPCR analysis, which showed that the expression levels of tRF-27-FDXXE6XRK45 were significantly higher in GC tissues than in the matched paracancerous tissues (P=0.0042) **(Fig. 1B)**. Meanwhile, we collected serum samples from GC patients and healthy donors, and RT-qPCR showed that the expression levels of tRF-27-FDXXE6XRK45 in the sera of GC patients were significantly higher than those of healthy donors (P<0.0001) **(Fig. 1C)**. In addition, we found the corresponding serum samples of these 20 GC tissues, and correlated the expression levels of tRF-27-FDXXE6XRK45 and discovered a good linear relationship** (Fig. 1D)**. Therefore, we selected tRF-27-FDXXE6XRK45 for our study.

### Basic information of tRF-27-FDXXE6XRK45

**Figure 2:**
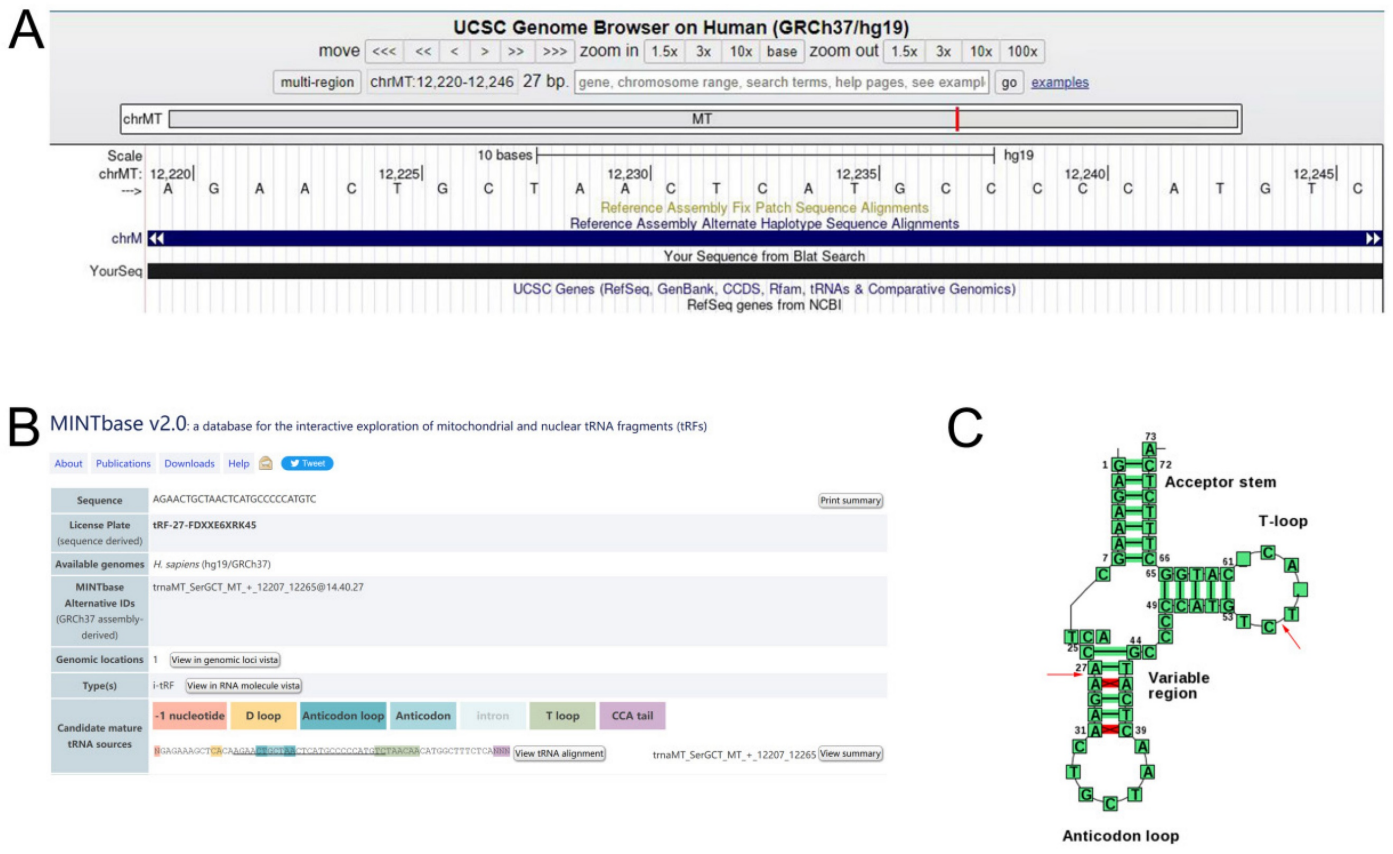
tRF-27-FDXXE6XRK45 is a type of i-tRF. **(A)** tRF-27-FDXXE6XRK45 is located on ChrMT with coordinates of 12220-12246 and a length of 27 bp. **(B)** tRF-27-FDXXE6XRK45 is an i-tRF with a length of 27 nucleotides (5'-AGAACTGCTAACTCATGCCCCCATGTC-3') in the MINTbase v2.0. **(C)** The cleavage site of tRF-27-FDXXE6XRK45 is located on the T-loop and Anticodon stem in tRNAdb.

We logged into the UCSC Genome Browser database (https://genome-asia.ucsc.edu/) and then opened the human genome build (GRCh37/hg19) for basic information of tRF-27-FDXXE6XRK45 and found that it originated from chromosome chrMT (12207-12265) with coordinates of 12220-12246 **(Fig. 2A)**. Using MINTbase v2.0 (https://cm.jefferson.edu/MINTbase/) and tRNAdb (http://trna.bioinf.uni-leipzig.de/DataOutput/Search), we determined that tRF-27- FDXXE6XRK45 is an i-tRF of 27 nucleotides in length (5'-AGAACTGCTAACTCATGCCCCCATGTC-3'), derived from trnaMT_SerGCT, with cleavage sites located on the T-loop and Anticodon stem **(Fig. 2B, C)**.

### Prerequisites for clinical applicability of tRF-27-FDXXE6XRK45

**Figure 3:**
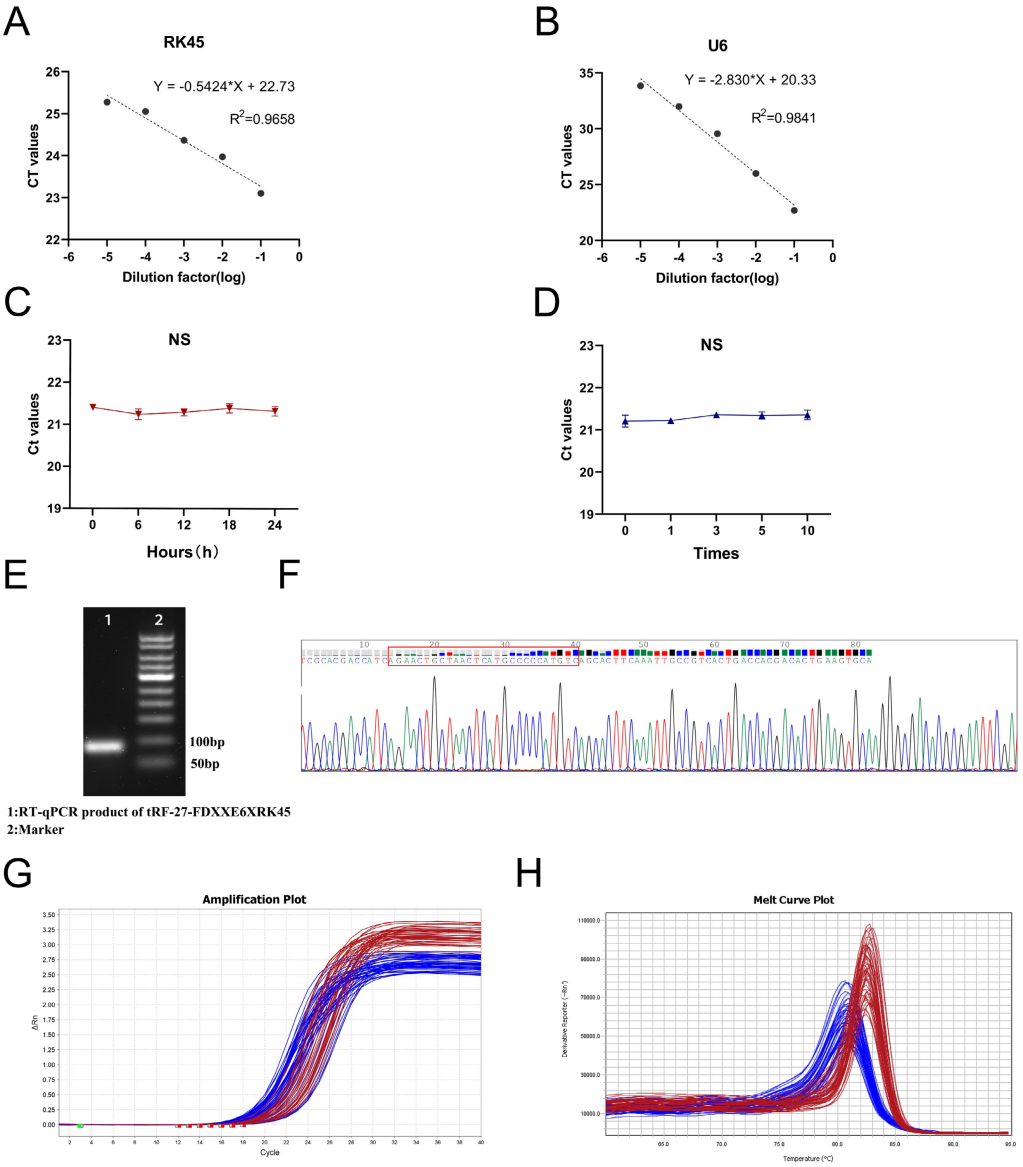
Evaluation of tRF-27-FDXXE6XRK45 detection methods. **(A, B)** Gradient dilution experiments showed good linearity. **(C, D)** Room temperature and repeated freezing and thawing experiments showed no significant change in the expression level of tRF-27-FDXXE6XRK45. **(E)** Agarose gel electrophoresis showed a single band of about 80 bp for the RT-qPCR product of tRF-27-FDXXE6XRK45. **(F)** Sanger sequencing of the qRT-PCR product confirmed that the product contained the full-length sequence of tRF-27-FDXXE6XRK45. **(G, H)** The amplification curve and melting curve of tRF-27-FDXXE6XRK45, the red lines represent tRF-27-FDXXE6XRK45, and the blue ones represent RNU6B.

To explore whether tRF-27-FDXXE6XRK45 has the potential to be used as a novel GC biomarker for clinical applications, we first performed a comprehensive evaluation of its detection methods. We analyzed detection accuracy using mixed serum and found that examination on tRF-27-FDXXE6XRK45 had good intra-assay and inter-assay coefficients of variation of 1.48% and 2.18%, respectively **(Table 1)**. Gradient dilution experiments also showed good linearity **(Fig. 3A, B)**. In addition, room temperature and repeated freeze-thawing experiments showed that tRF-27-FDXXE6XRK45 was resistant to external influences to some extent **(Fig. 3C, D)**, ensuring the reproducibility and stability of the assay. To ensure the integrity and accuracy of the RT-qPCR product, we conducted agarose gel electrophoresis experiments, and the development results showed clear individual bands of about 80 bp **(Fig. 3E)**. Meanwhile, Sanger sequencing showed that the RT-qPCR product contained the complete sequence of tRF-27-FDXXE6XRK45 **(Fig. 3F)** and was consistent with MINTbase v2.0. Additionally, the smooth amplification curve and single-peak specific melting curve also showed the specificity of the assay **(Fig. 3G, H)**. The above experimental results show that tRF-27-FDXXE6XRK45 meets the prerequisites for clinical applicability.

### Expression of tRF-27-FDXXE6XRK45 in GC sera and its clinical significance

**Figure 4:**
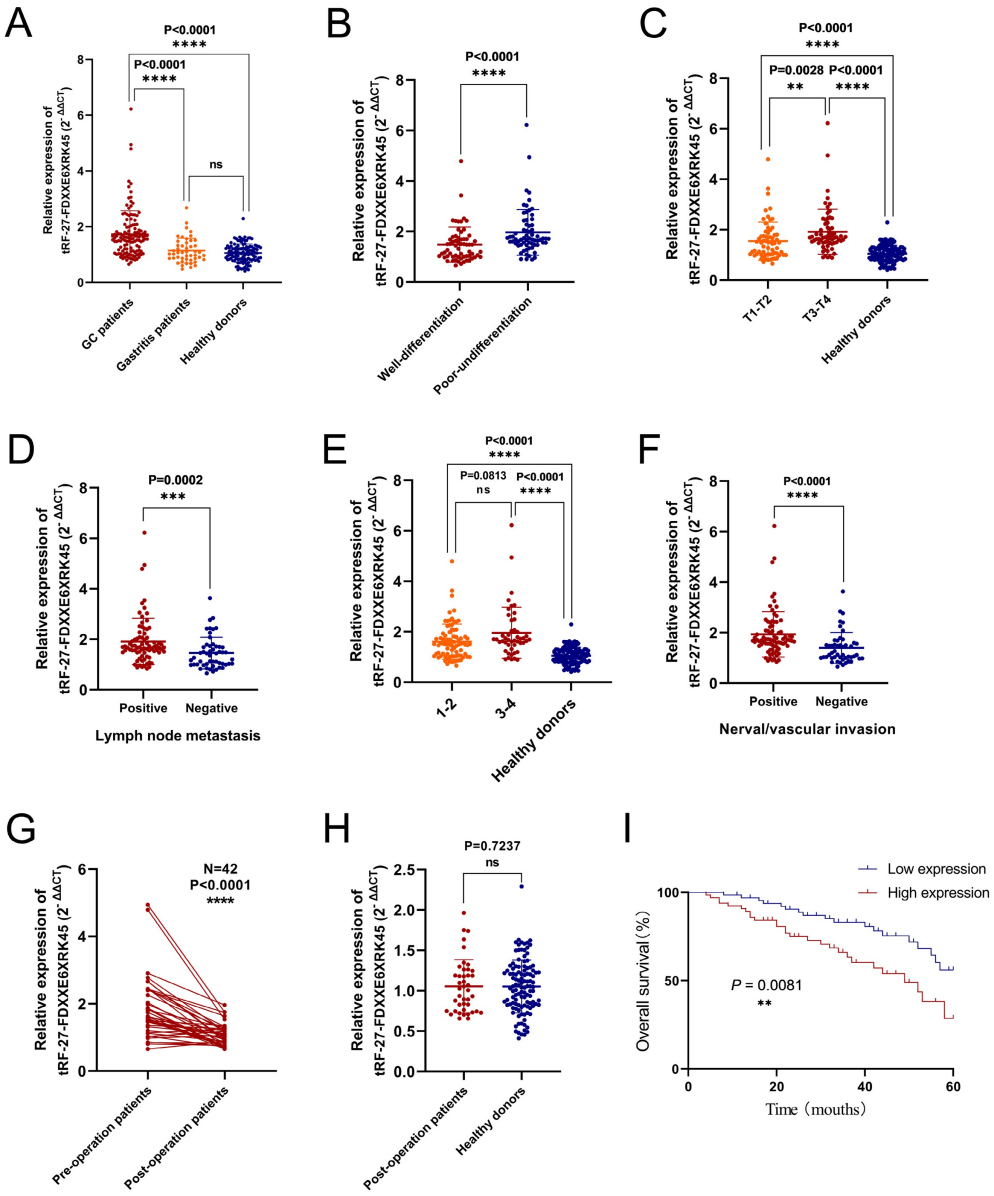
Diagnostic and prognostic value of serum tRF-27-FDXXE6XRK45. **(A)** The expression levels of tRF-27-FDXXE6XRK45 in serum samples from GC patients (n=130), gastritis patients (n=48), and healthy donors (n=115). **(B)** The expression levels of tRF-27-FDXXE6XRK45 in sera of the well (n=63) and poorly differentiated GC patients (n=67). **(C)** The expression levels of tRF-27-FDXXE6XRK45 in GC patients with different stages of the depth of tumor invasion and healthy donors (T1-T2: n=66; T3-T4: n=64; healthy donors: n=115). **(D)** The expression levels of tRF-27-FDXXE6XRK45 in sera of GC patients with (n=78) or without lymph node metastasis (n=52). **(E)** The expression levels of tRF-27-FDXXE6XRK45 in sera of stage Ⅰ-Ⅱ GC patients (n=83), stage Ⅲ-Ⅳ patients (n=47), and healthy donors (n=115). **(F)** The expression levels of tRF-27-FDXXE6XRK45 in sera of GC patients with (n=81) or without nerve/vascular invasion (n=49). **(G)** The expression levels of tRF-27-FDXXE6XRK45 in sera of 42 GC patients before and after surgery. **(H)** The expression levels of tRF-27-FDXXE6XRK45 in sera of postoperative GC patients and healthy donors. **(I)** Kaplan-Meier curve analysis of the relationship between the pre-operative expression level of tRF-27-FDXXE6XRK45 and the survival of GC patients. *P < 0.05; **P < 0.01; ***P < 0.001; ****P < 0.0001

To visualize the expression levels of tRF-27-FDXXE6XRK45 in GC sera, we collected serum samples from 130 GC patients, 48 gastritis patients, and 115 healthy donors. By RT-qPCR, we found that the expression levels of tRF-27-FDXXE6XRK45 in GC patients were significantly higher than those in gastritis patients (P<0.0001) and healthy donors (P<0.0001), while there was no significant difference in the expression levels of tRF-27-FDXXE6XRK45 in gastritis patients and healthy donors **(Fig. 4A)**. To explore the correlation between tRF-27-FDXXE6XRK45 expression levels and clinicopathological parameters of GC patients, we considered 65 GC patients with expression levels above the median as the high expression group and the others as the low expression group. Chi-square test results showed that tRF-27-FDXXE6XRK45 expression was significantly associated with the degree of differentiation (P=0.001), T stage (P=0.005), lymph node metastasis (P=0.001), TNM stage (P=0.018) and nerve/vascular invasion (P<0.0001), but not with age, sex, tumor size, Lauren classification, C-erbB-2, and MMR **(Table 2)**.

Next, we analyzed the differences in tRF-27-FDXXE6XRK45 expression levels by grouping clinicopathological parameters with significant correlation. We found that tRF-27-FDXXE6XRK45 expression levels were significantly higher in GC patients with poor differentiation than in GC patients with well-differentiation **(Fig. 4B)**. With the increase of tumor infiltration depth and lymph node metastasis, the expression levels of tRF-27-FDXXE6XRK45 also increased **(Fig. 4C, D)**. Subsequently, we divided GC patients into I-Ⅱ group and Ⅲ-Ⅳ group according to TNM stage and compared them with healthy donors. tRF-27-FDXXE6XRK45 expression levels were not significantly different in the I-Ⅱ group and Ⅲ-Ⅳ group (P=0.0813), but both were significantly higher than healthy donors (P<0.0001) **(Fig. 4E)**. Meanwhile, the expression levels of tRF-27-FDXXE6XRK45 were significantly higher in GC patients with vascular or neural invasion than in GC patients without invasion **(Fig. 4F)**.

To explore whether tRF-27-FDXXE6XRK45 could be a good serum marker to monitor the prognosis of GC patients, we followed up the postoperative sera tRF-27-FDXXE6XRK45 expression levels in 42 GC patients. It showed that most of them were reduced compared to preoperative (P<0.0001) **(Fig. 4G)**. Compared to sera from healthy donors, the tRF-27- FDXXE6XRK45 expression levels were not substantially different (P=0.7237) **(Fig. 4H)**. In addition, Kaplan-Meier analysis showed that GC patients with low pre-operative tRF-27-FDXXE6XRK45 expression levels had a higher survival rate than those with high pre-operative expression levels **(Fig. 4I)**. In conclusion, tRF-27-FDXXE6XRK45, with significantly elevated expression levels within the sera of GC patients, has the potential to be a biomarker for GC diagnosis and may help predict the prognosis of GC patients.

### Comparison and combination of tRF-27-FDXXE6XRK45 with other tumor markers

**Figure 5:**
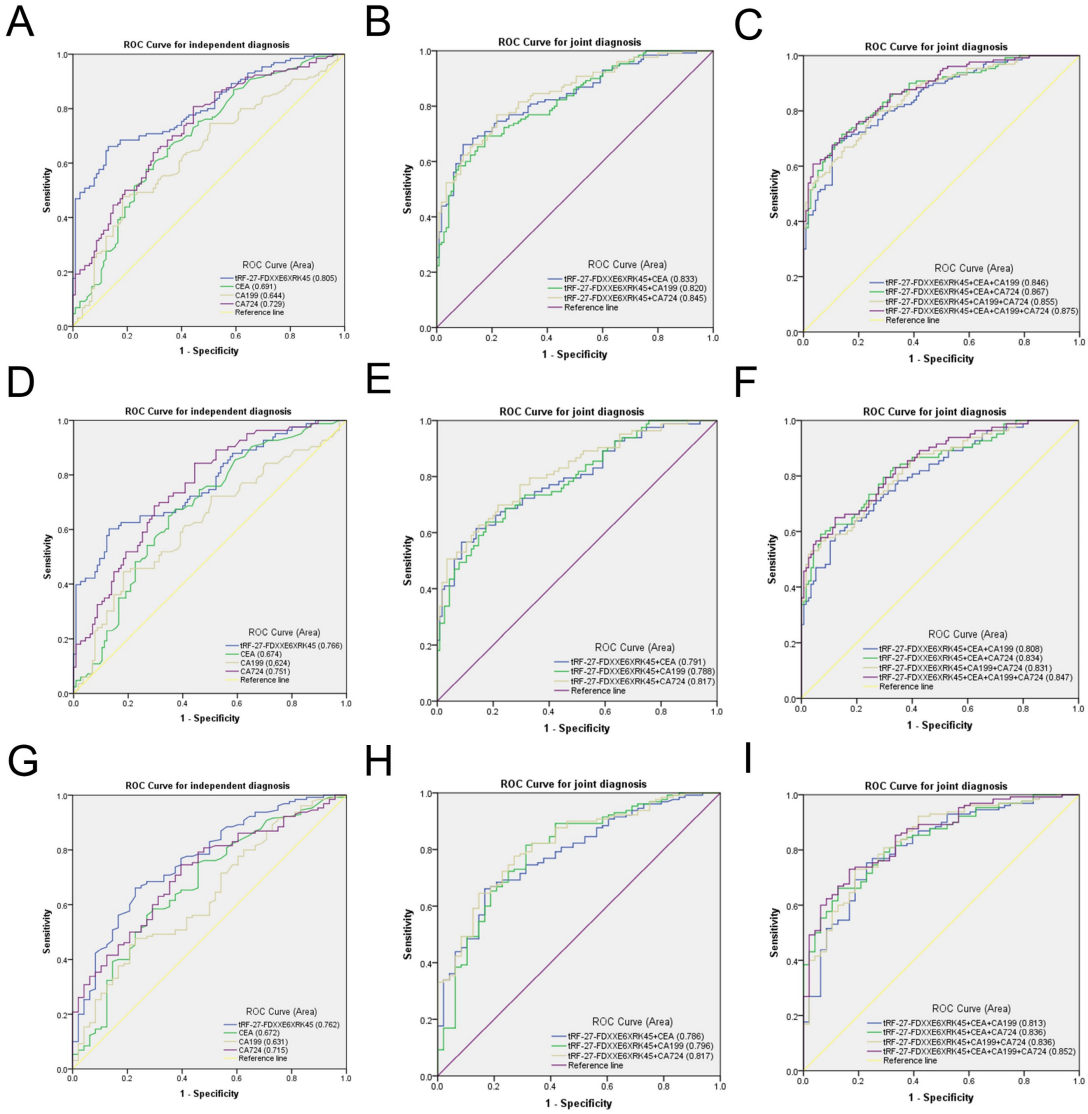
Evaluation of the diagnostic value of serum tRF-27-FDXXE6XRK45 in GC. **(A-C)** ROC analysis of tRF-27-FDXXE6XRK45, CEA, CA199, and CA724 in the independent and joint diagnosis of GC patients and healthy donors. **(D-F)** ROC analysis of tRF-27-FDXXE6XRK45, CEA, CA199, and CA724 in the independent and joint diagnosis of early GC patients and healthy donors. **(G-I)** ROC analysis of tRF-27-FDXXE6XRK45, CEA, CA199, and CA724 in the independent and joint diagnosis of GC and gastritis patients.

To evaluate the diagnostic efficacy of tRF-27-FDXXE6XRK45 as a GC diagnostic marker, we compared tRF-27-FDXXE6XRK45 with other GC markers. CEA, CA199, and CA724 are common GC diagnostic markers. We modeled ROC curves for the expression levels of tRF-27-FDXXE6XRK45, CEA, CA199, and CA724 in the sera of 130 GC patients and 115 healthy donors, and analyzed the area under the curve and found that the AUC of tRF-27-FDXXE6XRK45 was 0.805 (95% confidence interval (CI) 0.752-0.859), which was higher than 0.691 (95% CI 0.625-0.758) of CEA, 0.644 (95% CI 0.575-0.713) of CA199 and 0.729 (95% CI 0.667-0.792) of CA724 **(Fig. 5A)**. Meanwhile, when the cut-off point was 1.4362514485, and the Youden index was 0.5311, tRF-27-FDXXE6XRK45 had 66% SEN and 87% SPE in distinguishing GC patients from healthy donors. The SEN of CEA, CA199, and CA724 was 62%, 48%, and 55%; SPE was 68%, 81%, and 74%, respectively. In addition, the overall accuracy (ACCU), positive predictive value (PPV), and negative predictive value (NPV) of tRF-27-FDXXE6XRK45 were as follows: 76%, 85%, and 69%, higher than CEA, CA199, and CA724** (Table 3)**. tRF-27-FDXXE6XRK45, therefore, showed better diagnostic performance than other GC markers when analyzed alone. Next, tRF-27-FDXXE6XRK45 was combined with CEA, CA199, and CA724, respectively, and the AUC of the combined diagnosis was found to be higher than that of any single biomarker **(Fig. 5B)**; moreover, the diagnostic efficiency was further improved when three biomarkers were combined. The AUC reached the highest value of 0.875 (95% CI 0.834- 0.917) when all four biomarkers were combined **(Fig. 5C)**, and the SEN also reached the highest value of 96% **(Table 3)**. In conclusion, tRF-27-FDXXE6XRK45 is a potential GC marker, and its combination with other GC markers can improve diagnostic efficiency.

The high incidence and mortality of GC guide us to detect GC as early as possible. However, the low sensitivity of common GC markers CEA, CA199, and CA724 limits the early detection of GC 6. Then we urgently need to find a new GC marker with high sensitivity, certainly as high specificity as possible. We selected serum samples from 83 patients with early GC for detection. The ROC curve showed that the AUC of tRF-27-FDXXE6XRK45 was 0.766 (95% CI 0.698-0.834), which was better than that of CEA 0.674 (95% CI 0.599-0.748), CA199 0.624 (95% CI 0.544-0.704) and CA724 0.751 (95% CI 0.684-0.818) **(Fig. 5D)**. In addition, when the cut-off point was 1.4362514485 and the Youden index was 0.472, the SEN of tRF-27-FDXXE6XRK45 was 60%, SPE was 87%, ACCU, PPV, and NPV were 76%, 77%, and 75%, respectively, which were higher than CEA, CA199, and CA724 **(Table 4)**. Similarly, the AUC of the combination diagnosis was higher than that of the single biomarker in recognizing early GC patients and healthy donors **(Fig. 5E, F)**. The AUC reached the highest with the combination of the four biomarkers at 0.847 (95% CI 0.793-0.900), and the SEN increased to 95% **(Table 4)**. Therefore, tRF-27-FDXXE6XRK45 has potential value in the early diagnosis of GC.

Studies have found that chronic gastritis and GC have similar early symptoms, which are difficult to distinguish, so we need to identify gastritis and GC 26. We performed ROC analyses on 130 GC patients and 48 gastritis patients and found that the AUC of tRF-27-FDXXE6XRK45 was 0.762 (95% CI 0.684-0.839), which was higher than that of CEA 0.672 (95% CI 0.581-0.764), CA199 0.631 (95% CI 0.540-0.722) and CA724 0.715 (95% CI 0.635-0.795) **(Fig. 5G)**. When the cut-off point was 1.431740788, and the Youden index was 0.433, tRF-27-FDXXE6XRK45 had 66% SEN and 77% SPE in distinguishing GC patients from gastritis patients. ACCU, PPV, and NPV were 69%, 89%, and 46%, respectively, higher than CEA, CA199, and CA724 **(Table 5)**. When tRF-27-FDXXE6XRK45 was combined with other GC markers, the AUC increased **(Fig. 5H, I)**, reaching a maximum of 0.852 (95% CI 0.793-0.910), and SEN increased to 96% when all four GC markers were combined **(Table 5)**. In conclusion, we consider tRF-27-FDXXE6XRK45 as a potential biomarker to discriminate gastritis from GC.

### Downstream prediction of tRF-27-FDXXE6XRK45

**Figure 6:**
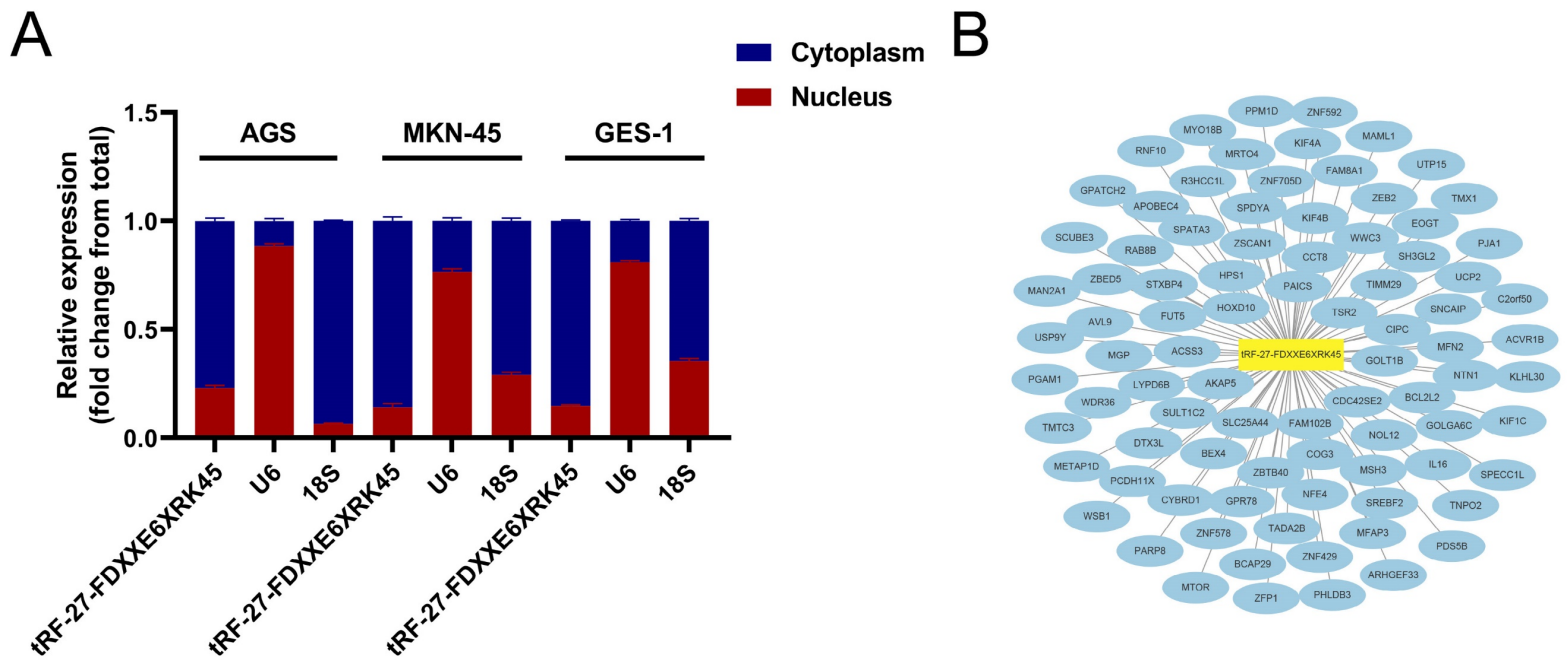
Prediction of tRF-27-FDXXE6XRK45 downstream. **(A)** Nuclear and cytoplasmic RNA isolation assay was performed on AGS, MKN-45, and GES-1 to detect tRF-27-FDXXE6XRK45. **(B)** Potential target genes of tRF-27-FDXXE6XRK45.

We used nuclear and cytoplasmic RNA isolation assays to visualize the distribution of tRF-27-FDXXE6XRK45 in MKN-45, AGS, and GES-1, showing that tRF-27-FDXXE6XRK45 is mainly concentrated in the cytoplasm **(Fig. 6A)**, which provides a general direction for our subsequent experiments. Next, we used miRanda, RNAhybrid, and TargetScan databases to find potential target sites of tRF-27-FDXXE6XRK45. The network diagram shows 87 target genes common to the three databases **(Fig. 6B)**, laying the foundation for further exploration of the mechanism of action of tRF-27-FDXXE6XRK45.

## Discussion

GC is a malignant disease of the gastrointestinal tract with a poor prognosis. Despite partial advances in early prevention and surgical treatments, GC remains the fifth most common cancer worldwide 4, 27. Since the early symptoms are not obvious 28, most patients cannot be diagnosed as GC in time. Therefore, early diagnosis has a great deal of clinical significance. Liquid biopsy, the critical tool for early detection and prevention of GC, is commonly used for its convenience. In recent years, this non-invasive examination method has gradually been more widely adopted clinically than invasive ones that are highly harmful, such as endoscopy 29. However, the diagnostic efficacy of current serum biomarkers for screening GC is low 30, so we are counting on finding a new, highly sensitive, and specific serum marker to improve the efficiency of early screening.

With the widespread popularity of high-throughput technologies, researchers have identified that more and more differentially expressed ncRNAs, such as circular RNAs, microRNAs, and long ncRNAs, play a significant role in cancers 31. tsRNAs, precursor or mature tRNA-derived RNA fragments, are highly enriched in body fluids and can be stably present 32, 33. Studies have found that tsRNAs have the potential as cancer biomarkers 34, 35. tsRNAs such as tsRNA-ValTAC-41 were found by Xue et al. to be novel biomarkers of pancreatic ductal adenocarcinoma and to play a potential functional role in cancer development 36. We believe that tsRNAs are involved in various biological regulatory pathways, providing new ideas for cancer diagnosis and treatment.

To search for a more effective GC biomarker, we performed high-throughput sequencing on three pairs of GC tissues and their paraneoplastic tissues, then validated them in GC tissues and sera, and selected tRF-27-FDXXE6XRK45, significantly different, for the follow-up study. According to MINTbase v2.0, tRF-27-FDXXE6XRK45 is an i-tRF derived from trnaMT_SerGCT with a length of 27 nucleotides. Evaluation of the assay showed that the stability and detection accuracy of tRF-27-FDXXE6XRK45 were in line with the premise of its clinical application. Subsequently, we examined the expression levels of tRF-27-FDXXE6XRK45 in more serum samples. We found that the levels were significantly higher in the sera of GC patients than in gastritis patients and healthy donors, implying that tRF-27-FDXXE6XRK45 could distinguish GC from gastritis and healthy individuals. In addition, tRF-27-FDXXE6XRK45 expression was significantly reduced in GC patients after surgery, even without significant differences from healthy individuals. Survival curves showed that high expression of tRF-27-FDXXE6XRK45 was associated with a low survival rate, suggesting that tRF-27-FDXXE6XRK45 can help monitor the prognosis of GC patients. More importantly, ROC curve analysis showed that tRF-27-FDXXE6XRK45 has higher diagnostic efficacy than CEA, CA199, and CA724, and the combined diagnosis can further improve the diagnostic value. Notably, the current biomarkers are not highly sensitive in the early diagnosis of GC, resulting in patients missing the optimal treatment time 6. Therefore, we conducted a diagnostic evaluation of early GC patients (stages I and Ⅱ). Surprisingly, ROC showed that tRF-27-FDXXE6XRK45 has a higher diagnostic efficiency than common clinical biomarkers, suggesting that it may have a great role in early diagnosis. In conclusion, we found that tRF-27-FDXXE6XRK45 has the potential to become a new GC biomarker.

Certainly, we need to investigate further the mechanism by which tRF-27-FDXXE6XRK45 affects GC progression. In papillary thyroid cancer, a 33nt tiRNA-Gly was significantly upregulated and promoted tumor cell proliferation and migration by binding to RBM17 37. Zhou et al. found that tsRNA-26576 could promote breast cancer cell proliferation, migration, and invasion and inhibit apoptosis by suppressing the expression of SPEN and FAT4 genes 38. Another study reported that tRF-33-Q1Q89P9L842205 was significantly downregulated in laryngeal squamous cell carcinoma, exerted inhibitory effects, and was associated with lymph node metastasis, clinical stage, and T-stage 39. Thus, tsRNAs can influence tumor progression by affecting cell growth, apoptosis, and migration. Similarly, we correlated tRF-27-FDXXE6XRK45 expression levels with clinicopathological parameters and found positive correlations with differentiation degree, T stage, TNM stage, lymph node metastasis, and nerve/vascular invasion. This suggests that tRF-27-FDXXE6XRK45 may be able to promote GC cell proliferation and metastasis, which provides ideas for a follow-up study.

Studies have shown that tsRNAs can interact with mRNA or protein to regulate gene expression. For example, under hypoxia, Goodarzi et al. found that tRFAsp-GTC and tRFGly-TCC produced by breast cancer cells competitively bind YBX1 and replace the 3′UTR in YBX1, resulting in reduced stability of endogenous oncogene transcripts, thereby inhibiting tumor cell proliferation 40. Shao et al. found that tRF-Leu-CAG plays a role similar to microRNAs in influencing NSCLC progression by regulating the expression of downstream target genes AURKA 41. These prompted us to further search for potential downstream targets of tRF-27-FDXXE6XRK45 and investigate its mechanism of action. Yan et al. found that mitochondrial miR-762 regulates the metabolism and mitochondrial function of cardiomyocytes by binding to endogenous NADH dehydrogenase subunit 2 42. Interestingly, according to MINTbase v2.0, tRF-27-FDXXE6XRK45 is derived from trnaMT_SerGCT. Mitochondrial tRNA-derived tsRNAs, just as tRF-27-FDXXE6XRK45, may have similar functions. Next, we will consider studying the effect of tRF-27-FDXXE6XRK45 on the metabolism and metastasis of GC cells, as well as the effect on mitochondrial function in GC cells. Furthermore, the results of nuclear and cytoplasmic RNA isolation assays showed that tRF-27-FDXXE6XRK45 was mostly present in the cytoplasm, which provided us with some thoughts for later research. In addition, we used three databases to predict the downstream target genes of tRF-27-FDXXE6XRK45 for the subsequent experiments.

It is concluded that tRF-27-FDXXE6XRK45 was significantly upregulated in GC sera and tissues and correlated with some pathological parameters, suggesting that a novel GC diagnostic marker could be mapped. The function and specific mechanism of tRF-27-FDXXE6XRK45 in GC remain investigated and may serve as a future therapeutic target.

## Conclusion

We revealed for the first time that the expression levels of tRF-27-FDXXE6XRK45 were substantially upregulated in GC tissues and sera, by which tRF-27-FDXXE6XRK45 can differentiate GC patients from gastritis patients and healthy donors. Furthermore, tRF-27-FDXXE6XRK45, associated with GC progression and its expression levels decreased after GC surgery, can be used for prognostic monitoring of GC patients. Compared with traditional GC biomarkers, tRF-27-FDXXE6XRK45 has a higher diagnostic value. Next, we will further explore the mechanism of tRF-27-FDXXE6XRK45.

## Figures and Tables

**Table 1:** The Intra-Assay CV and the Inter-Assay CV of tRF-27-FDXXE6XRK45.

	tRF-27-FDXXE6XRK45	RNU6B
Intra-assay CV, %	1.48	1.63
Inter-assay CV, %	2.18	2.25

CV, coefficient of variation.

**Table 2:** Clinicopathological analysis of tRF-27-FDXXE6XRK45.

Parameter		No. of patients	tRF-27-FDXXE6XRK45(high)	tRF-27-FDXXE6XRK45(low)	P-value
Sex	Male	88	42	46	0.453
	Female	42	23	19	
Age(year)	<60	31	15	16	0.837
	≥60	99	50	49	
Tumor size	<5	90	41	49	0.128
	≥5	40	24	16	
Differentiation grade	Well-differentiated	63	22	41	0.001
	Poorly-undifferentiated	67	43	24	
T stage	T1-T2	66	25	41	0.005
	T3-T4	64	40	24	
Lymph node status	Positive	78	48	30	0.001
	Negative	52	17	35	
TNM stage	Ⅰ-Ⅱ	83	35	48	0.018
	Ⅲ-Ⅳ	47	30	17	
Nerve/vascular invasion	Positive	81	53	28	<0.0001
	Negative	49	12	37	
Lauren classification	Intestinal type	49	21	28	0.120
	Mixed type	38	17	21	
	Diffuse type	43	27	16	
C-erbB-2	Positive	8	6	2	0.144
	Negative	122	59	63	
MMR	dMMR	8	5	3	0.465
	pMMR	122	60	62	

**Table 3:** The diagnostic performance of tRF-27-FDXXE6XRK45, CEA, CA199, and CA724 in differentiating GC patients from healthy donors.

	SEN,%	SPE,%	ACCU,%	PPV,%	NPV,%
tRF-27-FDXXE6XRK45	0.66(86/130)	0.87(100/115)	0.76(186/245)	0.85(86/101)	0.69(100/144)
CEA	0.62(80/130)	0.68(78/115)	0.64(158/245)	0.68(80/117)	0.61(78/128)
CA199	0.48(62/130)	0.81(93/115)	0.63(155/245)	0.74(62/84)	0.58(93/161)
CA724	0.55(71/130)	0.74(85/115)	0.64(156/245)	0.70(71/101)	0.59(85/144)
tRF-27-FDXXE6XRK45+CEA	0.85(111/130)	0.61(70/115)	0.74(181/245)	0.71(111/156)	0.79(70/89)
tRF-27-FDXXE6XRK45+CA199	0.82(106/130)	0.70(81/115)	0.76(187/245)	0.76(106/140)	0.77(81/105)
tRF-27-FDXXE6XRK45+CA724	0.84(109/130)	0.65(75/115)	0.75(184/245)	0.73(109/149)	0.78(75/96)
tRF-27-FDXXE6XRK45+CEA+CA199	0.92(120/130)	0.48(55/115)	0.71(175/245)	0.67(120/180)	0.85(55/65)
tRF-27-FDXXE6XRK45+CEA+CA724	0.94(122/130)	0.44(51/115)	0.71(173/245)	0.66(122/186)	0.86(51/59)
tRF-27-FDXXE6XRK45+CEA+CA199+CA724	0.96(125/130)	0.35(40/115)	0.67(165/245)	0.63(125/200)	0.89(40/45)

SEN, sensitivity; SPE, specificity; ACCU, overall accuracy; PPV, positive predictive value; NPV, negative predictive value.

**Table 4:** The diagnostic performance of tRF-27-FDXXE6XRK45, CEA, CA199, and CA724 in differentiating early GC patients from healthy donors.

	SEN,%	SPE,%	ACCU,%	PPV,%	NPV,%
tRF-27-FDXXE6XRK45	0.60(50/83)	0.87(100/115)	0.76(150/198)	0.77(50/65)	0.75(100/133)
CEA	0.59(49/83)	0.68(78/115)	0.64(127/198)	0.57(49/86)	0.70(78/112)
CA199	0.45(37/83)	0.81(93/115)	0.66(130/198)	0.63(37/59)	0.67(93/139)
CA724	0.59(49/83)	0.74(85/115)	0.68(134/198)	0.62(49/79)	0.71(85/119)
tRF-27-FDXXE6XRK45+CEA	0.81(67/83)	0.61(70/115)	0.69(137/198)	0.60(67/112)	0.81(70/86)
tRF-27-FDXXE6XRK45+CA199	0.77(64/83)	0.70(81/115)	0.73(145/198)	0.65(64/98)	0.81(81/100)
tRF-27-FDXXE6XRK45+CA724	0.80(66/83)	0.65(75/115)	0.71(141/198)	0.62(66/106)	0.82(75/92)
tRF-27-FDXXE6XRK45+CEA+CA199	0.89(74/83)	0.48(55/115)	0.65(129/198)	0.55(74/134)	0.86(55/64)
tRF-27-FDXXE6XRK45+CEA+CA724	0.93(77/83)	0.44(51/115)	0.65(128/198)	0.55(77/141)	0.89(51/57)
tRF-27-FDXXE6XRK45+CEA+CA199+CA724	0.95(79/83)	0.35(40/115)	0.60(119/198)	0.51(79/154)	0.91(40/44)

SEN, sensitivity; SPE, specificity; ACCU, overall accuracy; PPV, positive predictive value; NPV, negative predictive value.

**Table 5:** The diagnostic performance of tRF-27-FDXXE6XRK45, CEA, CA199, and CA724 in differentiating GC patients from gastritis patients.

	SEN,%	SPE,%	ACCU,%	PPV,%	NPV,%
tRF-27-FDXXE6XRK45	0.66(86/130)	0.77(37/48)	0.69(123/178)	0.89(86/97)	0.46(37/81)
CEA	0.62(80/130)	0.65(31/48)	0.62(111/178)	0.82(80/97)	0.38(31/81)
CA199	0.48(62/130)	0.77(37/48)	0.56(99/178)	0.85(62/73)	0.35(37/105)
CA724	0.55(71/130)	0.73(35/48)	0.60(106/178)	0.85(71/84)	0.37(35/94)
tRF-27-FDXXE6XRK45+CEA	0.85(111/130)	0.46(22/48)	0.75(133/178)	0.81(111/137)	0.54(22/41)
tRF-27-FDXXE6XRK45+CA199	0.82(106/130)	0.63(30/48)	0.76(136/178)	0.85(106/124)	0.56(30/54)
tRF-27-FDXXE6XRK45+CA724	0.84(109/130)	0.58(28/48)	0.77(137/178)	0.84(109/129)	0.57(28/49)
tRF-27-FDXXE6XRK45+CEA+CA199	0.92(120/130)	0.33(16/48)	0.76(136/178)	0.79(120/152)	0.62(16/26)
tRF-27-FDXXE6XRK45+CEA+CA724	0.94(122/130)	0.35(17/48)	0.78(139/178)	0.80(122/153)	0.68(17/25)
tRF-27-FDXXE6XRK45+CEA+CA199+CA724	0.96(125/130)	0.25(12/48)	0.77(137/178)	0.78(125/161)	0.71(12/17)

SEN, sensitivity; SPE, specificity; ACCU, overall accuracy; PPV, positive predictive value; NPV, negative predictive value.

## Abbreviations

GC: Gastric cancer
CEA: Carcinoembryonic antigen
CA: Carbohydrate antigen
SEN: Sensitivity
SPE: Specificity
ncRNAs: Non-coding RNAs
tRNAs: Transfer RNAs
tsRNAs: tRNA-derived small RNAs
tRFs: tRNA-derived fragments
tiRNAs: tRNA halves
RT-qPCR: RNA quantitative reverse transcription PCR
ROC: Receiver operating characteristic
AUC: Area under curve
CV: coefficient of variation
CI: Confidence interval
ACCU: Overall accuracy
PPV: Positive predictive value
NPV: Negative predictive value
